# Utilization of point-of-care tests among general practitioners, a cross-sectional study

**DOI:** 10.1186/s12875-022-01643-9

**Published:** 2022-03-09

**Authors:** Ricarda Oehme, Angelika Sabine Sandholzer-Yilmaz, Marcus Heise, Thomas Frese, Thomas Fankhaenel

**Affiliations:** Institute of General Practice and Family Medicine, Martin-Luther University Halle-Wittenberg, Magdeburger Straße 8, 06112 Halle (Saale), Germany

**Keywords:** Point-of-care-testing, Laboratory, General practice, Utilitzation, Estimated usefulness, Germany

## Abstract

**Background:**

Point-of-care testing (POCT) has numerous potential benefits to improve health care service, especially in resource-limited settings. We aim to identify which POC-tests (POCTs) of laboratory parameters are known, employed, and rated as useful by general practitioners (GPs).

**Methods:**

A questionnaire with 27 POCTs was posted to a random selection of GPs (*n* = 451) in Saxony, Germany.

**Results:**

A total of 208 GPs replied (response rate 46.1%). Out of 27 POCTs, each GP knew an average of 20.3 as laboratory parameters and 9.2 as POCTs. Urine test strips (99.0%), blood glucose test (98.1%), and Troponin I/T (86.4%) were the best-known, followed by INR/Quick (82.5%), Microalbumin (79.1%), and D-dimer (78.6%) POCTs. Yet, solely 0 to 13 POC tests were actually used (mean value 4.6). Urine test strips were employed most frequently (97.6%), followed by blood glucose test (94.7%), Troponin I/T (57.8%), Microalbumin (57.3%), and INR/Quick POCTs (41.7%). Heart fatty binding protein (H-FABP), Syphilis, Coeliac disease, and Malaria appeared as the least frequently used POCTs. The majority of the GPs declared 14 of the 27 POCTs to be useful.

**Discussion/conclusion:**

The most recurrently employed POCTs are those for diagnosing or monitoring diabetes mellitus, ensued by POCTs addressing acute cardiovascular diseases (Troponin I/T, D-dimer) or monitoring the therapy of infectious diseases or the anticoagulant therapy. POCTs most often rated as useful by GPs are also widely known and frequently used. Nonetheless, the majority of GPs rate only a very limited number of POCTs as useful. Frequent concerns might be low economic benefit, over-reliance, and test accuracy coming along with the complex implementation of the tests requiring technical skills, accurate storage, and the correct interpretation of test results.

**Trial registration:**

In accordance with the (Model) Professional Code for Physicians in Germany, neither human body materials nor data that can be assigned to a specific human being are used in our study. A declaration of no objection from the Ethics Committee of the Martin-Luther University Halle-Wittenberg (Medical Faculty) confirms no professional or ethical concerns due to completely anonymized data collection and analysis. Our study was therefore not registered in a corresponding registry.

**Supplementary Information:**

The online version contains supplementary material available at 10.1186/s12875-022-01643-9.

## Background

Point-of-care testing (POCT) has excellent potential to enable practitioners to make more reliable decisions and to perform the appropriate intervention more promptly [1, 2].

POCT endeavors to bring testing closer to the patient. At the time of patient consultation, it allows to generate fast results and therefore supports the efficiency of clinical decision-making. One result is a benefit on morbidity and mortality rates [1, 3]. This approach can enhance the satisfaction of both, the general practitioner (GP) and the patient [1, 4–6].

During the past decades, the quantity of parameters that POCT can assess and the uptake in primary care settings has rapidly expanded worldwide [2, 7, 8].

The advantages of POCTs are particularly evident in the current COVID-19 pandemic. Compared to PCR testing in a laboratory, the use of POCT led to a reduction of time until the results were available and also led to an improvement in infection control [9].

The choice of POCTs is wide. Present-day includes laboratory tests with small or large measuring devices, portable equipment for ECG, ultrasound, spirometry, etc. All provide point-of-care diagnostic tests—even in the patient's home. In this study only POC tests for laboratory parameters with small portable devices are considered, which are appropriate for home visits.

Common reasons for consulting a family doctor’s practice are urinary tract infections (UTI) [10]. In addition to the regular anamneses, using a urine testing strip evaluates up to 10 different markers and can help to deliver the diagnosis of urinary tract infection or injury, kidney disease, diabetes, and its complications [11].

Worldwide, the prevalence of Diabetes mellitus type 2 is increasing [12]. Within the past years, POCTs for Blood glucose and HbA1c helped to diagnose diabetes and to monitor treatment response [13, 14]. POCTs for Mircoalbumin may also indicate diabetes associated complications (e.g. kidney damage) [15].

To detect acute dangerous conditions, various POCTs are available. Troponin T, for example, is one of the primary markers in diagnosing critically myocardial infarction. That also applies to NT-proBNP for heart failure, glucose testing for hypo- and hyperglycaemic causes, and D-dimer POCTs to exclude pulmonary embolism and deep vein thrombosis. It is furthermore suitable for INR to monitor the therapy of patients who are taking Vitamin K-antagonists like Marcumar. Concerning respiratory tract infections, the CRP value proved to reach a significant reduction of an unnecessary prescription of antibiotics, which is then associated with avoiding the development of antibiotic resistance [16, 17]. Procalcitonin POCT, on the other hand, is intended to facilitate the differentiation between viral and bacterial respiratory tract infections with regard to the consideration of an antibiotic therapy and is also suitable for the monitoring progress [18].

However, the availability of new POCTs also challenges physicians to efficiently use and interpret them [5]. Besides, the benefits must outweigh the expenses [19].

In international studies and despite these clear advantages, only a limited quantity of POCTs are estimated as valuable in clinical practice [5, 20, 21].

Approximately 10 years ago, we set out a cross-sectional survey to evaluate the use of POCTs in the federal state of Saxony. At that time, this study aimd to find out whether POC tests are already established in the daily practice of general practitioners. Questioning whether the parameters and the associated POC tests are known, which ones are considered useful and which tests are used. In addition, we wanted to find out differences in the use of POCTs (e.g. age, gender, regional structures (city/rural) or activity as GPs who teach undergraduates) [22]. The surveyed general practitioners only judged the frequently employed POCTs as relevant. At that time, POCT was mere of insignificant importance as a diagnostic tool and was commonly known and used to monitor diabetes and hypertension, as well as associated complications through Urine testing strips, Blood glucose, and Microalbumin. They have also been employed to identify acute conditions (Troponin, D-dimer), and to test for infections (Influenza, CRP) and pregnancy (human chorionic gonadotropin). Notwithstanding, the amount of POC-test that GPs judged as useful was low. This is independent of the age and gender of the GPs, whether they are teaching undergraduates or not, and the location of the practice (metropolitan or small-town rural). Although rural arean physicians appear to be better informed about POC testing (for HFABP, CRP, PSA, Heliocobacter pylori, and Hydrogen breath test), there are no differences in frequency of use or importance [22].

We performed the current survey to examine the changes in POCTs application of GPs in Saxony, focusing on usage, perceived usefulness, and prominence of multiple POCTs.

## Methods

### Make-up of the questionnaire

The base for the structure of the representative questionnaire was an equivalent study from 10 years ago. With the assistance of a GP, a psychologist, and a general practice trainee, this qualified questionnaire was self-designed. The 27 POCTs recorded in the aforementioned earlier study were revised.. PubMed, as well as familiar search engines like Google, and also online suppliers of laboratory supplies, e.g. https://www.praxisdienst.de, have been used for the revision [23].

We selected small and transportable POCTs, that are easily applicable, do not require any additional equipment, and can also be used outside the practice, for example during a home consultation. A further criterium was that the results had to be instantly available (within a few seconds up to 30 min). An easy sample acquisition and processing (using whole blood, urine, saliva, etc.), and uncomplicated storage of materials was also required. For example, due to the large measurement device, we did not select HBA1c POCT.

Based on the above criteria, we included some new POCTs evaluating infectious diseases like Malaria, Syphilis, HIV, and Procalcitonin, such as cardiovascular POCT CK-MB and also POCTs to examine different drugs like INR/Quick for the efficiency of anticoagulation therapy as well as one, not furtherly described Drugtest. BNP was replaced by a NT-proBNP POCT. Obsolete tests have been replaced, namely the Urea breath test, Hydrogen breath test, M2-Pyruvate kinase test, MRSA, Albumin, H1N1 flu, and Salmonella typhii test. A Hydrogen breath test to detect lactose intolerance, for example, is not usable during the home visit because of too long measuring time by five breath tests with a special breath measure test device.

The two-page questionnaire included sociodemographic questions and appraisals about the use and the practicality of 27 POCTs. For each of the 27 tests, four binary questions had to be answered: concerning familiarity of the laboratory parameter, interpretation of this parameter, knowledge about the corresponding POCTs, and whether these POCTs are used. Also, respondents were asked to rate the utility of these tests on a four-point Likert scale: “very useful”, „rather useful “, „rather not useful “,or “not useful”.

A prepaid return envelope, as well as a cover letter declaring the background and the purpose of the study, were attached to each questionnaire.

The questionnaire is available in the appendix.

### Sample and survey

The sample selection was based on the register of members of the Kassenärztliche Vereinigung Sachsen (Saxon Association of Statutory Health Insurance Physicians KVS, https://www.kvs-sachsen.de; December 2019). In Saxony, the group consisted of 2706 GPs, including specialists for general medicine, internists, and general practitioners without specialist training (“Praktischer Arzt / Ärztin”). To achieve a sufficiently large response rate of 200 to 300 responses, we aimed to write to at least 450 of the 2706 GPs. Thus, we selected a sample of *n* = 451 GPs, which corresponds to every 6th GP in Saxony. Using a function of the computer program "Microsoft Excel", from 2706 numbers, corresponding to the alphabetically ordered and consecutively enumerated GPs in the list, 451 coincidental numbers were generated. A new lineup of 451 randomly selected GPs appeared. In January 2020, these 451 GPs received the questionnaire in written form. Each potential participant was contacted one time, no pre-warnings or reminders were used. The attendees did not receive any incentives for replying to the questionnaire.

### Statistical analysis

To obtain descriptive statistics and in order to analyze the characteristics of the study sample, we used absolute and relative frequencies as well as means and standard variations. By employing a one-sample proportion test, sociodemographic differences between the population and the sample have been examined. Discrepancies were considered statistically significant for *p* < 0.05. The 95%-confidence intervals have also been determined for these comparisons. In order to compare the POCTs in terms of utilization and knowledge, we illustrated the aggregated assessments of the GPs in bar charts (percentages of GPs who use or are familiar with a test). Spearman’s rank correlation coefficient was used to distinguish associations between these accumulated evaluations.

The estimated utility of multiple POCTs was dichotomized in two categories: “(rather) not useful” and “(rather) useful”. Corresponding percentages of GPs, who consider various POCTs as (rather) useful, were depicted in bar charts. All analyses were performed using SPSS Version 25.0 [24].

## Results

### Sample description

A total of 451 questionnaires have been sent by mail and 208 have been answered. Due to incomplete replies (a refusal to answer almost every part), two of these questionnaires had to be excluded from further analysis. None of the questionnaires was undeliverable for the reason of incorrect address data. According to the AAPOR standard definition RR2, this consequently issues a response rate of 45.7% [25].

The sociodemographic characteristics of the sample are displayed in Table 1. It consists of a majority of women (64.6%) and has an average age of 52.1 years. These characteristics match the population's distributions. A large part of our individuals operates in solo practices (60.7%), with an average of 18.8 years in practice. In our sample, compared to the population, there are more respondents with academic degree (overrepresentation, *p* = 0.006) and fewer respondents without medical specialty training (practical physicians) (underrepresentation, *p* = 0.024).

**Table 1:** Sociodemographic description of responding and total of GPs in Saxony

	**Sample (*n* = 206)**			**Population statistics (i = 2690)**		
	**n / n**_**valid**_	**%**	**95% C.I**	**n / n**_**valid**_	**%**	***P***
Gender						
Male	73/206	35.4		1074/2690	39.9	0.187
Female	133/206	64.6		1616/2690	60.1	
Age in years (categorized)						
Under 35	4/200	2.0		50/2690	1.9	0.912
35 to < 40	17/200	8.5		225/2690	8.4	0.959
40 to < 50	62/200	31.0		689/2690	25.6	0.08
50 to < 60	68/200	34.0		913/2690	33.9	0.976
60 to < 66	34/200	17.0		566/2690	21.0	0.165
66 and older	15/200	7.5		247/2690	9.2	0.406
Age in years (M ± SD); *n* = 200	52.1	± 10.3	[50.7; 53.5]	53.4	± 10.2	0.076
Academic degree						
Yes ^a)^	129/204	63.2		1451/2706	53.6	0.006
No	75/204	36.8		1255/2706	46.4	
Medical specialisation						
General practitioner	134/206	65.0		1666/2690	61.9	0.36
Internist	68/206	33.0		892/2690	33.2	0.951
None ^b)^	3/206	1.5		132/2690	4.9	0.024
General practitioner & Internist	1/206	0.5				
Practice structure						
Solo practice	122/201	60.7			n.a	
Group practice ^c)^	79/201	39.3			n.a	
Additional qualification ^d)^						
Yes	91/206	44.2			n.a	
No	115/206	55.8			n.a	
Years in practice (M ± SD); *n* = 202	18.8	± 11.7			n.a	
Teaching undergraduates						
Yes	31/187	16.60			n.a	
No	156/187	83.40			n.a	

*p* one-sample proportion test, *n.a.* this information could not be researched for comparison

Data of the population are from the KVS of 10.06.2020 with 2690 GPs listed at this time, except academic degree. These comparative data sources our own research of 12/2019 with a population of 2706 GPs

^a^By an academic degree is meant a habilitation (for a professorship) or a doctorate (for a PhD). No academic degree: including German diploma not shown here separately

^b^Practical physician´ / ´physician without specialist medical training

^c^Practice with more than one GP including community practice (economic and organizational association), shared practice (common practice rooms, but without forming an economic unit), and medical care centre

^d^e.g. emergency or palliative medicine, acupuncture, diabetology, and endocrinology

### Knowledge and utilization of POCTs

**Fig. 1:**
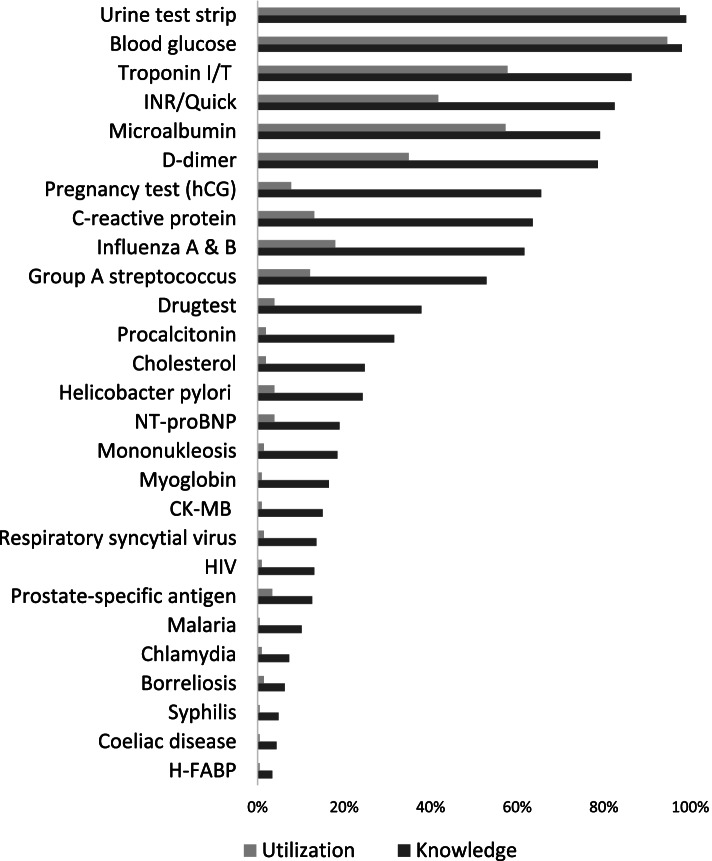
Knowledge (black) und utilization (grey) of POCTs among GPs in Saxony (*n* = 206)

The questionnaire filed 27 parameters and their relating POCTs. Aggregated replies concerning familiarity and utilization of the 27 POC-tests are presented in Fig. 1. On average, of the 27 parameters reported, primary care physicians knew 20.3 as laboratory parameters and only 9.2 as POCTs. Urine test strips (99.0%), blood glucose test (98.1%), and Troponin I/T (86.4%) were the best known, followed by INR/Quick (82.5%), Microalbumin (79.1%), and D-dimer (78.6%) POCTs. Nevertheless, solely 0 to 13 POC tests were actually used (mean value 4.6). Urine test strips were employed most frequently (97.6%), followed by blood glucose tests (94.7%), Troponin I/T (57.8%), Microalbumin (57.3%), and INR/Quick POCTs (41.7%).

Our results further indicate that known POCTs are also used more frequently. Between utilization and knowledge based on the aggregated proportions, we find a nonparametric correlation of *ρ* = 0.94; *p* < 0.01.

### Estimated usefulness of POCTs

**Fig. 2:**
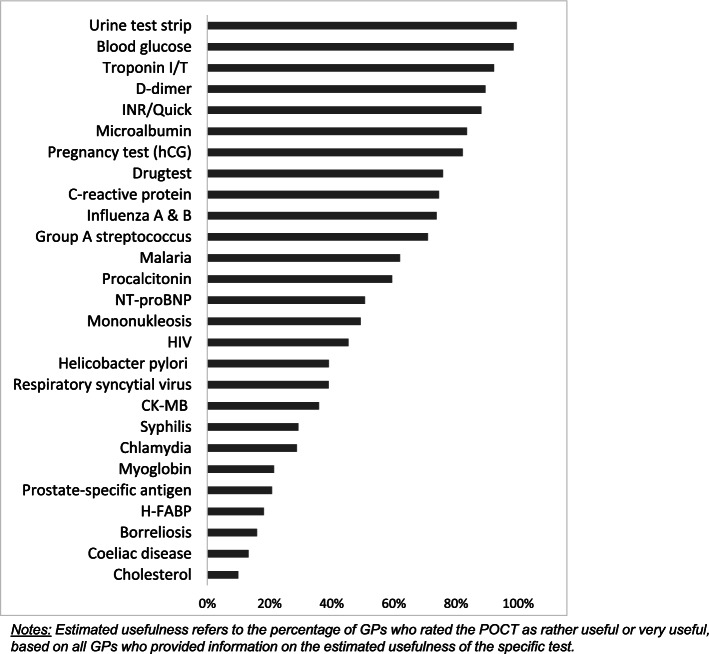
Estimated usefulness of POCTs (*n* = 206)

The estimated usefulness of POCTs as rather useful or very useful by GPs is demonstrated in Fig. 2. The most frequently considered useful POCTs were Urine test strips (99.5%), Blood glucose (98.5%), Troponin (92.3%), D-dimer (89.5%), and INR/Quick (88.2%). As least useful were ranked Cholesterol (9.9%), Coeliac disease (13.2%), Borrelia (16%) and H-FABP (18.2%) POCTs. At a rate of more than 50% of the respondents, 14 out of 27 POCTs rated as "very useful" or "rather useful" (as cumulative percentages in Fig. 2), and four of them as “very useful” (Blood glucose 92.0%, Urine test strips 91.8%, Troponin I/T 68.5% and INR/Quick 65.7%, not shown in Fig. 2).

The results of POCTs considered most useful largely replicate the findings respecting knowledge and utilization of POCTs.

## Discussion

In this research, we wanted to analyze the knowledge and utilization of POCTs among GPs in Saxony and compare the results to data that was collected 10 years ago and to available international data. We observed that GPs know numerous available POCTs but only employ a few of them in daily practice. Moreover, well-known and regularly used POCTs formed a coherent cluster with POCT considered most useful. These results underline an association between estimated usefulness, knowledge and utilization of POCTs. In fact, we observe a strong association between usefulness and knowledge (*ρ* = 0.87; *p* < 0.01) as well as between usefulness and utilization of POCTs (*ρ* = 0.82; *p* < 0.01). These results largely replicate the previous findings in Fig. 1 respecting knowledge and utilization of POCTs. That means, POCTs that are widely known and frequently used are also the ones that are commonly appraised as valuable by GPs. POCTs to diagnose and monitor Diabetes are the most regularly practiced.

### Comparison current and previous results of POCTs to international data

Due to a random selection from the register of members of the KVS, the sample selection for both studies was done with the same method [22].

Comparing the involved POCTs among the two studies, both included a list of 27 POCTs. Nevertheless, some tests were no longer part of the current study (see method section).

Between the survey carried out almost 10 years ago and the present study, there were some similarities in terms of knowledge, utilization, and estimated usefulness of available and known POCTs [22]. In both surveys, the most famous and most frequently used POCTs were Urine testing strips, Blood glucose, Microalbumin, and Troponin. INR/Quick POCT, which was newly indicluded in the current survey, was also frequently known (82.5%) and used (41.7%), followed by D-dimer, Pregnancy test, CRP, Influenza A & B, and Group A streptococcus POCTs.

This seems to be quite similar to the previous survey 10 years ago and to international data. According to a 2016 study by Sohn et al., US family physicians use POCTs to diagnose diabetes mellitus, urinary tract infections, strep throat, influenza, pregnancy, anemia, infectious mononucleosis, anticoagulation, acute cardiac conditions, and lipid disorders [13].

In general practice, numerous POCTs for diagnosing and monitoring cardiovascular diseases exist, for example, Troponin I/T, NT-proBNP, INR/Quick, D-dimer, and H-FABP. Whereas the majority of the GPs appreciate and use Troponin I/T, D-dimer, and INR/Quick. Within the present survey, it is noteworthy that the D-dimer POCT presents a big difference between knowledge (78.6%) and utilization (35.0%). If the probability is low, D-dimer testing can be helpful to exclude a diagnosis of deep vein thrombosis or pulmonary embolism [20, 26]. Reasons could be concerns about the test accuracy. A positive test result does not have to be thrombosis but can also have other causes so that it is not of much use to the GP in making a diagnosis. NT-proBNP and H-FABP are rarely known and used, in our current survey, as well as in the previous. Some studies are evaluating the efficiency in detecting myocardial damage of patients with acute chest pain, comparing the measurement of high sensitive Troponin I/T and H-FABP. Assessments revealed that the diagnostic value of H-FABP POCT is rather meager and inferior to Troponin I/T POCT [27, 28]. NT-proBNP POCT having been part during the previous survey as BNP POCT, has proven to result in earlier diagnosis, reduced hospitalizations, and seem to be cost-effective for diagnosing and controlling heart failure [29, 30]. However, in our present study, this POCT was rarely known (18.9%) and rarely used (3.9% of GPs). Solely 50.7% of the GPs judged it as useful. One possible reason for the rather poor evaluation could be the cautious recommendations of the AWMF National Health Care Guideline for Chronic Heart Failure of 10/2019. Due to their lower sensitivity, compared to the corresponding laboratory tests, POCTs for BNP and NT-proBNP are not suitable for the exclusion of heart failure without additional transthoracic echocardiography [31].

POCTs for diagnosing infections have become increasingly important for general practice. There exist various POCTs for infectious diseases. We included C-reactive protein, Influenza A and B, Group A streptococcus, Procalcitonin, Helicobacter pylori, Mononukleosis, Respiratory syncytial virus, HIV, Malaria, Chlamydia, Borreliosis, and Syphilis (Fig. 1).

In the prior and the present research, the majority of the GPs knew about the possibility of CRP POCTs. However, only 13.1% currently reported using them (Fig. 1). In a Dutch study, experts favored the CRP POCTs over its laboratory equivalent, as the POCTs allow an instantaneous decision regarding the prescription of antibiotic treatment. 80% of the Dutch GPs declare to use CRP POCTs on a regular basis [5, 32]. CRP POCTs may reduce antibiotic prescribing at the index visit, but there is a higher rate of return visits [33]. In other words, everything has side effects, even POCTs.

Since the questionnaire was sent before the COVID-19 pandemic, it would certainly be interesting to learn whether the present use of CRP and PCT POCTs has increased at first COVID symptoms, such as cough or fever. In addition, PCT POCT has been covered by health insurance since July of 2018. Throughout the next few years, it will probably show whether the current use (just under 2% in our survey) will be increasing due to the recent reimbursement of costs.

Sexually transmitted diseases are still very common in Germany and are associated with significant morbidity and mortality worldwide [34, 35]. There exist multiple POCTs for diagnosing and therapy monitoring. Chlamydia, which was already part of the previous survey, HIV, and Syphilis POCTs, which were newly included, showed low familiarity and usage rate. Only HIV POCT were rated as useful by 45.4% of GPs. These tests offer the advantage of a prompt diagnosis, allowing immediate treatment and a reduction of disease transmission [36]. In our current survey, POCT is strongly recommended in German guidelines and is expected to increase in the future [37, 38].

### Possible limitations of the POCTs utilization in general practice

GPs have common concerns on the reliability of the POCTs and the comparability to central laboratory results, over-reliance on tests, usage without appropriate indication, and uncertain use and interpretation [1]. Besides, very often the staff needs to be trained and/ or taught about the handling of the POCT. The relatively meager billing option might be an additional argument why German physicians do not employ them [39]. Influenza POCTs, for example, are generally recommended by the Robert Koch Institute but are not reimbursed by German legal health insurances [40].

Some of the POCTs require an appropriate storage which might be costly. An additional concern of the GPs is that POCTs do not guarantee an improved patient outcome [1, 5, 21].

An inference of Deutsches Ärzteblatt from 2017 stated that POCTs should only be employed with extreme caution. Limitations in terms of sensitivity, specificity, and cost-effectiveness should always be weighed against the available quality-assured laboratory diagnostics [41]. As most POCTs have not been sufficiently evaluated, Schols et al. also recommend that GPs should remain critical of which tests they order [1].

Throughout the usage of POCTs, the practice location seems to play a role. The previous survey affirmed a difference in the knowledge, but not regarding the utilization of POCTs between rural GPs and their urban colleagues. As a result, we did not newly investigate this [22]. A study from the UK did not reveal any correlation concerning the demographic data neither [22, 42]. However, this could play a role in resource-limited environments [43].

### Strength and limitations

The current study examines a coincidental sample from a limited list of all registered general practitioners in Saxony. The representativeness criterion and the connection with the satisfactory response rate of 46.1% can be seen as a strength of the present survey. Yet, to obtain more responses a timely reminder would have been advantageous. The sample solely represents 208 of the 2706 GPs in Saxony—representing 7.6% of the target population in only one geographical area in Germany. That, being said, could be considered a weakness.

The self-administered pen-and-pencil questionnaire was modified and improved several times. Eventually, there was a pre-test by a GP in private practice without complaints. For comparability, we employed a tried and tested questionnaire with a commonly intelligible structure similar to the one ten years ago, so we abstained from a pilot-test.

In addition, there is evidence of an overrepresentation of respondents with an academic dregee and an underrepresentation of GPs without specialist medical training. Possible motives for these biases in response behavior are workload within the practice, specific interest in the topic of the survey, or in university research in general. These characteristics are presumed confounders with regards to the usage of POCTs and impose the relevant limitations on the generalizations of our results.

Impacts on social usefulness, e.g., an over-reporting on the level of knowledge and utilization of POCTs, cannot be excluded. Bias from some inadequately completed questionnaires is also possible. Some laboratory or POC tests were totally disregarded, leaving entire lines, and even rarely, entire sections unfilled, so-called unit non-response. Whether the GPs were merely oblivious of the POCTs or deliberately did not answer cannot be differentiated. Altogether, we excluded 2 of these inadequately completed questionnaires from the analysis. Also, we connot differentiate how often respondents use the POCT when they indicate using it. In addition, we included a 4-point Likert scale where participants could choose if a POCT was (very) useful or not (very) useful. There existed no other response options such as „I am unsure “. The use of only four categories limits the validity. Compared to the previous survey, the current representation solicited different GPs. Therefore, longitudinal comparisons on familiarity and usage of POCTs have analogous limitations. Moreover, the present questionnaire did not investigate the clinical syndromes for which the POCTs are used. As POCTs are most likely to be considered useful in the context of diabetes, this creates a further constraint [42].

The questionnaire did not examine motives why the GPs might estimate the tests as rather not utile, nor did it investigate possible concerns that might prevent GPs from using the existing POCTs. In order to improve the utilization of POCTs in general practice, further research should assess this. Due to the heterogeneous health systems, diverse limitations may arise.

Another significant characteristic affecting the employment of POCTs involves their size and portability; particularly with respect to the distinction between bedside and near-bedside POCTs [44]. The present questionnaire only considered portable POCTs with small devices, which are appropriate for home visits. Yet, further research should approach the utilization and the perceived utility of near-bedside POCTs.

## Conclusions

POCTs are expected to improve clinical practice as they are designed to be easily applicable. They provide instant results and allow making immediate clinical decisions, which helps to improve the effectiveness, especially in ambulant settings. However, the actual utilization in daily practice involves mainly a few tests for diagnosing and monitoring diabetes, determining urinary tract and other infections, and filtering out acute cardiac syndromes. For general practitioners, many POCTs are not useful as they test chronic or rather innocuous conditions. Some POC tests are more specialized and better suited for primary care internists. GPs, therefore, employed few but useful tests to support their practice. It is a resource-conserving use of POCT. Ultimately, our study reveals that GPs are unwilling to adopt technologies that are not beneficial to patient care or the profitability of their practices.

## Supplementary Information

**Additional file 1.**

## Data Availability

All data, generated or analyzed during this study, are included in this published article [and its supplementary information files].

## References

[1] Schols A, Dinant G-J, Cals JW (2018). Point-of-care testing in general practice: just what the doctor ordered?. Br J Gen Pract.

[2] Shephard M, Shephard A, Matthews S, Andrewartha K (2020). The Benefits and Challenges of Point-of-Care Testing in Rural and Remote Primary Care Settings in Australia. Arch Pathol Lab Med.

[3] Schols AMR, Dinant G-J, Hopstaken R, Price CP, Kusters R, Cals JWL (2018). International definition of a point-of-care test in family practice: a modified e-Delphi procedure. Fam Pract.

[4] Laurence CO, Gialamas A, Bubner T, Yelland L, Willson K, Ryan P, Beilby J (2010). Patient satisfaction with point-of-care testing in general practice. Br J Gen Pract.

[5] Kip MMA, Hummel JM, Eppink EB, Koffijberg H, Hopstaken RM, IJzerman MJ, Kusters R (2019). Understanding the adoption and use of point-of-care tests in Dutch general practices using multi-criteria decision analysis. BMC Fam Pract.

[6] Price CP (2001). Point-of-care testing. Impact on medical outcomes. Clin Lab Med.

[7] Luppa PB, Müller C, Schlichtiger A, Schlebusch H (2011). Point-of-care testing (POCT): Current techniques and future perspectives. Trends Analyt Chem.

[8] St John A, Price CP (2014). Existing and Emerging Technologies for Point-of-Care Testing. Clin Biochem Rev.

[9] Brendish NJ, Poole S, Naidu VV, Mansbridge CT, Norton NJ, Wheeler H (2020). Clinical impact of molecular point-of-care testing for suspected COVID-19 in hospital (COV-19POC): a prospective, interventional, non-randomised, controlled study. Lancet Respir Med.

[10] Kranz J, Schmidt S, Lebert C, Schneidewind L, Mandraka F, Kunze M (2018). The 2017 Update of the German Clinical Guideline on Epidemiology, Diagnostics, Therapy, Prevention, and Management of Uncomplicated Urinary Tract Infections in Adult Patients: Part 1. Urol Int.

[11] Schot MJC, van Delft S, Kooijman-Buiting AMJ, de Wit NJ, Hopstaken RM (2015). Analytical performance, agreement and user-friendliness of six point-of-care testing urine analysers for urinary tract infection in general practice. BMJ Open.

[12] Roth GA, Abate D, Abate KH, Abay SM, Abbafati C, Abbasi N (2018). Global, regional, and national age-sex-specific mortality for 282 causes of death in 195 countries and territories, 1980–2017: a systematic analysis for the Global Burden of Disease Study 2017. The Lancet.

[13] Sohn AJ, Hickner JM, Alem F (2016). Use of Point-of-Care Tests (POCTs) by US Primary Care Physicians. J Am Board Fam Med.

[14] Gialamas A, St John A, Laurence CO, Bubner TK (2010). Point-of-care testing for patients with diabetes, hyperlipidaemia or coagulation disorders in the general practice setting: a systematic review. Fam Pract.

[15] Lloyd M, Kuyl J, Jaarsveld H (2014). Evaluation of point-of-care tests for detecting microalbuminuria in diabetic patients. South African Family Practice.

[16] Cooke J, Llor C, Hopstaken R, Dryden M, Butler C. Respiratory tract infections (RTIs) in primary care: narrative review of C reactive protein (CRP) point-of-care testing (POCT) and antibacterial use in patients who present with symptoms of RTI. BMJ Open Respir Res. 2020.10.1136/bmjresp-2020-000624. 10.1136/bmjresp-2020-000624 PMC7476490 32895246

[17] Ward C (2018). Point-of-care C-reactive protein testing to optimise antibiotic use in a primary care urgent care centre setting. BMJ Open Qual.

[18] Bublak R. Leitartikel: Der Procalcitonin-Test – für Hausärzte labormedizinische Ausgeburt sondergleichen. Springer Medizin Verlag GmbH, Ärzte Zeitung. 7.5.2018. Available from: https://www.aerztezeitung.de/Medizin/Der-Procalcitonin-Test-fuer-Hausaerzte-labormedizinische-Ausgeburt-sondergleichen-299407.html. Accessed 4 Mar 2022.

[19] Drain PK, Hyle EP, Noubary F, Freedberg KA, Wilson D, Bishai WR (2014). Diagnostic point-of-care tests in resource-limited settings. Lancet Infect Dis.

[20] Howick J, Cals JWL, Jones C, Price CP, Plüddemann A, Heneghan C (2014). Current and future use of point-of-care tests in primary care: an international survey in Australia, Belgium, The Netherlands, the UK and the USA. BMJ Open.

[21] Jones CHD, Howick J, Roberts NW, Price CP, Heneghan C, Plüddemann A, Thompson M (2013). Primary care clinicians' attitudes towards point-of-care blood testing: a systematic review of qualitative studies. BMC Fam Pract.

[22] Frese T, Steger K, Deutsch T, Schmid GL, Sandholzer H (2016). Use of point-of-care tests among general practitioners: a cross-sectional study in Saxony. Germany Rural Remote Health.

[23] Praxisdienst. PoC Tests & medizinische Schnelltests. Available from: https://www.praxisdienst.de/Laborbedarf/Tests/PoC+Tests/. Accessed 24 Aug 2019.

[24] IBM Corp. IBM SPSS Statistics for Windows, Version 25.0. Released 2017. Armonk, NY: IBM Corp.

[25] The American Association for Public Opinion Research. Standard Definitions: Final Dispositions of Case Codes and Outcome Rates for Surveys. Revised edition. AAPOR. 2016. Available from: https://www.aapor.org/aapor_main/media/publications/standard-definitions20169theditionfinal.pdf. Accessed 4 Mar 2022.

[26] El Tabei L, Holtz G, Schürer-Maly C, Abholz H-H (2012). Accuracy in diagnosing deep and pelvic vein thrombosis in primary care: an analysis of 395 cases seen by 58 primary care physicians. Dtsch Arztebl Int.

[27] Willemsen RT, Winkens B, Kietselaer BL, Smolinska A, Buntinx F, Glatz JF, Dinant G-J (2019). Evaluating possible acute coronary syndrome in primary care: the value of signs, symptoms, and plasma heart-type fatty acid-binding protein (H-FABP). A diagnostic study BJGP Open.

[28] Kellens S, Verbrugge FH, Vanmechelen M, Grieten L, van Lierde J, Dens J (2016). Point-of-care heart-type fatty acid binding protein versus high-sensitivity troponin T testing in emergency patients at high risk for acute coronary syndrome. Eur Heart J Acute Cardiovasc Care.

[29] Bugge C, Sether EM, Pahle A, Halvorsen S, SonboKristiansen I (2018). Diagnosing heart failure with NT-proBNP point-of-care testing: lower costs and better outcomes. A decision analytic study. BJGP Open.

[30] Shimizu N, Kotani K (2020). Point-of-care testing of (N-terminal pro) B-type natriuretic peptide for heart disease patients in home care and ambulatory care settings. Pract Lab Med.

[31] Bundesärztekammer (BÄK), Kassenärztliche Bundesvereinigung (KBV), Arbeitsgemeinschaft der Wissenschaftlichen Medizinischen Fachgesellschaften (AWMF). Nationale VersorgungsLeitlinie Chronische Herzinsuffizienz – Langfassung, 3. Auflage. Version 3. 2019. Available from: https://www.leitlinien.de/themen/herzinsuffizienz/3-auflage/kapitel-3. Accessed 06 Mar 2022.

[32] Kip MMA, Noltes AM, Koffijberg H, IJzerman MJ, Kusters R (2017). Improving early exclusion of acute coronary syndrome in primary care: the added value of point-of-care troponin as stated by general practitioners. Prim Health Care Res Dev.

[33] Martínez-González NA, Keizer E, Plate A, Coenen S, Valeri F, Verbakel JYJ (2020). Point-of-Care C-Reactive Protein Testing to Reduce Antibiotic Prescribing for Respiratory Tract Infections in Primary Care: Systematic Review and Meta-Analysis of Randomised Controlled Trials. Antibiotics (Basel).

[34] Bremer V, Dudareva-Vizule S, Buder S, der Heiden M an, Jansen K. Sexuell übertragbare Infektionen in Deutschland. Bundesgesundheitsbl. 2017;60:948–57. 10.1007/s00103-017-2590-1. 10.1007/s00103-017-2590-1 28741188

[35] World Health Organization. Sexually transmitted infections (STIs). 11.02.2021. https://www.who.int/news-room/fact-sheets/detail/sexually-transmitted-infections-(stis). Accessed 12 Feb 2021.

[36] Huntington SE, Burns RM, Harding-Esch E, Harvey MJ, Hill-Tout R, Fuller SS (2018). Modelling-based evaluation of the costs, benefits and cost-effectiveness of multipathogen point-of-care tests for sexually transmitted infections in symptomatic genitourinary medicine clinic attendees. BMJ Open.

[37] Meyer T, Schüttler CG, Straube E, Roß RS, Stürmer M, Jansen K (2017). Schnelltest-Diagnostik sexuell übertragbarer Infektionen in niedrigschwelligen Einrichtungen : Gemeinsame Stellungnahme des RKI, PEI und der DSTIG. Bundesgesundheitsblatt Gesundheitsforschung Gesundheitsschutz.

[38] Arbeitsgemeinschaft der Wissenschaftlichen Medizinischen Fachgesellschaften (AWMF). Langfassung der Leitlinie Sexuell übertragbare Infektionen (STI) – Beratung, Diagnostik und Therapie. 03.08.2018. Available from: https://www.awmf.org/leitlinien/detail/ll/059-006.html.

[39] Hausen T. Warum wird der CRP-Test nicht besser honoriert? Der Hausarzt 16/2020. 06.10.2020. Available from: https://www.hausarzt.digital/praxis/ebm/warum-wird-der-crp-test-nicht-besser-honoriert-75209.html. Accessed 12 Feb 2021.

[40] Lange W, Uphoff H. Influenza-Diagnose: Zur Praktikabilität von Schnelltests. Dtsch Arztebl 2002; 99(8): A-481 / B-386 / C-364. Available from: https://www.aerzteblatt.de/archiv/30530/Influenza-Diagnose-Zur-Praktikabilitaet-von-Schnelltests. Accessed 06 Mar 2022.

[41] Orth M. Schnelltests: Mit Bedacht einsetzen. Dtsch Arztebl 2017;114(17):[13]. 10.3238/PersInfek.2017.04.28.04.

[42] Turner PJ, van den Bruel A, Jones CHD, Plüddemann A, Heneghan C, Thompson MJ (2016). Point-of-care testing in UK primary care: a survey to establish clinical needs. Fam Pract.

[43] Mashamba-Thompson TP, Sartorius B, Drain PK (2018). Operational assessment of point-of-care diagnostics in rural primary healthcare clinics of KwaZulu-Natal, South Africa: a cross-sectional survey. BMC Health Serv Res.

[44] Larsson A, Greig-Pylypczuk R, Huisman A (2015). The state of point-of-care testing: a European perspective. Ups J Med Sci.

