# Supplementary material for: Utilization of point-of-care tests among general practitioners, a cross-sectional study
Source: BMC Prim Care. 2022 Mar 9;23:41. doi: 10.1186/s12875-022-01643-9 (PMC8906527; doi:10.1186/s12875-022-01643-9)
Supplement: Supplementary file 1 — Additional file 1. [file 12875_2022_1643_MOESM1_ESM.pdf]

Angaben zur Person

Facharzt für: O Allgemeinmedizin, seit ______
O Innere Medizin, seit ______
O Sonstige: ___________ , seit ______

Zusatzbezeichnung (bitte alle nennen): ____

akademischer Grad: O Habilitation
O Promotion
O Diplom
O keiner

Alter: ________

Geschlecht: O männlich
O weiblich

Tätigkeit: O Einzelpraxis
O Gemeinschaftspraxis
O Praxisgemeinschaft
O MVZ

Mitgliedschaften: O SGAM
O DEGAM
O Hausärzteverband
O keine
O andere: ______________________

Lehrarzt einer Universität: O nein O ja, Universität ______________________

Welche der folgenden diagnostischen Schnelltests nutzen Sie in Ihrer Praxis? Bitte Zutreffendes ankreuzen.

Schnelltest	Mir ist der Labor-parameter bekannt.	Mir ist die Anwendung/ Interpretation des Parameters bekannt.	Mir ist der Schnelltest bekannt.	Ich nutze diesen Schnelltest.	Ich halte diesen Test für …			
					sehr sinnvoll	eher sinnvoll	eher nicht sinvoll	nicht sinnvoll
Urin Teststreifen	O	O	O	O	O	O	O	O
Mikroalbumin im Urin	O	O	O	O	O	O	O	O
Schwangerschaftstest (hCG)	O	O	O	O	O	O	O	O
Blutzucker	O	O	O	O	O	O	O	O
Cholesterin	O	O	O	O	O	O	O	O
D-Dimer	O	O	O	O	O	O	O	O
CRP	O	O	O	O	O	O	O	O
Procalcitonin (PCT)-Test	O	O	O	O	O	O	O	O
INR/Quick Sofortmessung	O	O	O	O	O	O	O	O
Troponin I/T	O	O	O	O	O	O	O	O

Rückfax an 0345/ 557 5340

Schnelltest	Mir ist der Labor-parameter bekannt.	Mir ist die Anwendung/ Interpretation des Parameters bekannt.	Mir ist der Schnelltest bekannt.	Ich nutze diesen Schnelltest.	Ich halte diesen Test für …			
					sehr sinnvoll	eher sinnvoll	eher nicht sinvoll	nicht sinnvoll
Myoglobin	O	O	O	O	O	O	O	O
CK-MB	O	O	O	O	O	O	O	O
Heart fatty binding protein (hFABP)	O	O	O	O	O	O	O	O
NT-pro BNP	O	O	O	O	O	O	O	O
Influenza A und B -Test	O	O	O	O	O	O	O	O
Mononukleose (EBV-Ak)	O	O	O	O	O	O	O	O
Respiratory Syncytial Virus (RSV)	O	O	O	O	O	O	O	O
Streptokokken A	O	O	O	O	O	O	O	O
Borrelien-Schnelltest (Borreliose-Ak)	O	O	O	O	O	O	O	O
Chlamydien	O	O	O	O	O	O	O	O
Syphilis (Treponema pallidum-Ak)	O	O	O	O	O	O	O	O
Helicobacter pylori	O	O	O	O	O	O	O	O
Zöliakie-Gluten-Test	O	O	O	O	O	O	O	O
Prostata spezifisches Antigen (PSA)	O	O	O	O	O	O	O	O
HIV	O	O	O	O	O	O	O	O
Drogenschnelltest	O	O	O	O	O	O	O	O
Malaria	O	O	O	O	O	O	O	O
